# Efficacy of Concurrent Training in Breast Cancer Survivors: A Systematic Review and Meta-Analysis of Physical, Psychological, and Biomarker Variables

**DOI:** 10.3390/healthcare13010033

**Published:** 2024-12-27

**Authors:** Ricardo Madeira, Dulce Esteves, Adriana Maia, Ana R. Alves, Diogo L. Marques, Henrique P. Neiva

**Affiliations:** 1 Department of Sport Sciences, University of Beira Interior, Convento de Santo António, 6201-001 Covilhã, Portugal; ricardomadeira94@gmail.com (R.M.); desteves@ubi.pt (D.E.); adriana.maia1999@gmail.com (A.M.); asra@ubi.pt (A.R.A.); diogo.marques@ubi.pt (D.L.M.); 2 Research Center in Sports Sciences, Health Sciences and Human Development (CIDESD), Convento de Santo António, 6201-001 Covilhã, Portugal; 3 Health Sciences Research Center, University of Beira Interior (CICS-UBI), Av. Infante D. Henrique, 6201-506 Covilhã, Portugal

**Keywords:** cancer, exercise, strength, cardiorespiratory fitness, concurrent training

## Abstract

Background: Breast cancer treatments often cause serious side effects, but physical exercise has shown the potential to improve both the physical and psychological health outcomes of survivors. This review and meta-analysis aimed to synthesize and analyze the scientific evidence on the effectiveness of concurrent training on physical, psychological, and biomarkers variables on breast cancer survivors.; Methods: A systematic review and meta-analysis was registered in PROSPERO (CRD42024571851). The ISI Web of Science, PubMed, and Scopus databases were searched. The methodological quality of all the included studies was assessed using the Cochrane risk of bias tool. This review included 14 articles that met the inclusion criteria on the effect of concurrent training on breast cancer survivors. Results: The results of the meta-analysis on body composition revealed a significant overall effect on body mass (effect size [ES] = −2.23; 95% CI: −4.16, −0.29) and body mass index (ES = −0.66; 95% CI: −1.32, 0.01). In contrast, no significant differences were shown in the % fat mass (ES = −2.63; 95% CI: −5.58, 0.33). Strength significantly improved after simultaneous training (ES = 4.93; 95% CI: 1.94, 7.92). In addition, cardiorespiratory fitness (maximum oxygen consumption) showed significant improvements after simultaneous training (ES = 3.03; 95% CI: 1.88, 4.19). Conclusions: The research shows that concurrent training, including strength and aerobic exercises, promotes significant improvements in body mass, body mass index, muscle strength, and cardiorespiratory fitness. However, the effectiveness of the training depends on the intensity, duration and frequency of the exercise, as well as the individualization of the programs.

## 1. Introduction

Breast cancer is the most common type of cancer in women globally and was responsible for 2 million cases and 700,000 deaths by 2020 [1]. Approximately 78% of all cancer diagnoses occur in adults aged 60 and older [2]. Factors, such as genetics, environment, and lifestyle choices—including obesity, smoking, aging, and sedentary behavior—contribute to cancer development and reduce treatment effectiveness [3,4,5,6,7,8].

Treatment for breast cancer typically includes surgery, adjuvant chemotherapy, radiotherapy, and endocrine therapies [9]. While these treatments improve survival rates, they also lead to side effects [10,11,12]. These can include fatigue, sarcopenia, osteopenia, cardiovascular diseases, weight gain, immunosuppression, inflammation, and sleep disturbances [13,14,15,16]. The evaluation of biomarkers is vital to understanding the protective effects of exercise, guiding personalized interventions and improving the diagnosis, treatment, and quality of life of cancer patients [17,18]. Elevated levels of testosterone and C-reactive protein (CRP) have been associated with an increased risk of breast cancer, whereas higher levels of sex hormone-binding globulin (SHBG) correlate with a reduced risk [17,18]. Conversely, obesity is linked to insulin resistance and hyperinsulinemia, leading to increased insulin-like growth factor I production (IGF-I) [17]. These metabolic changes stimulate ovarian androgen synthesis and inhibit SHBG production, resulting in elevated testosterone levels and enhanced estradiol bioavailability, thereby increasing the risk of breast cancer [18].

Consequently, the literature indicates a significant growth in oncological exercise, with increasing evidence supporting its role in mitigating the adverse effects of cancer treatments [5]. Studies show that physically active individuals have a 20% to 40% lower risk of developing cancer and a 24% reduced risk of cancer recurrence [19,20]. Regular physical exercise, particularly aerobic and/or resistance training among female breast cancer survivors, has shown improvements in several health parameters [19,21,22,23,24,25]. These include increased muscle strength and endurance, improved cardiopulmonary fitness, reduced abdominal circumference, maintenance of bone mineral density, increased lean body mass, and decreased levels of fatigue and anxiety [19,21,22,23,24,25]. Given the varied benefits of both resistance and aerobic training, there has been scientific interest in combining these two forms of exercise in the same session. This type of training, which combines strength and aerobic exercises to simultaneously promote gains in strength and aerobic capacity, is known as concurrent training [26]. However, there is currently no consensus regarding the optimal strategy for concurrent training prescription [26]. Randomized controlled trials on breast cancer survivors have suggested that concurrent training may significantly improve quality of life, reduce depression and fatigue, positively impact body composition, and enhance physical fitness [27,28,29].

Nevertheless, despite the growing number and quality of clinical trials examining the impact of concurrent training on breast cancer survivors, research remains limited [30] and somewhat inconclusive regarding the benefits of different configurations of the acute training variables (e.g., relative intensity [29]) on health outcomes. Therefore, in this review and meta-analysis, we aim to consolidate and evaluate scientific evidence on the effectiveness of concurrent training on physical, psychological, and biomarker variables, as well as its potential adverse effects in this population. This review aims to contribute to theoretical discourse and provide recommendations for future research in this field. The two main research questions arising from this review are as follows: (1) What are the physiological effects of concurrent training in individuals with breast cancer? (2) What are the psychological effects of concurrent training in individuals with breast cancer? Additionally, the study will explore the effects of concurrent training in more common biomarkers in individuals with breast cancer due to their crucial role in the diagnosis and treatment of breast cancer.

## 2. Materials and Methods

### 2.1. Review Registration

This systematic literature review was carried out following the Preferred Reporting Items for Systematic Reviews and Meta-Analyses (PRISMA) guidelines and was registered on PROSPERO (registry number: CRD42024571851).

### 2.2. Search Strategy and Eligibility Criteria

The article search strategy included using the ISI Web of Knowledge, Scopus, and PubMed databases to identify relevant scientific articles published between 2000 and July 2024. The search specifically targeted studies examining the effects of concurrent training performed within the same session on individuals diagnosed with breast cancer. Keywords employed in the search included those listed individually and/or in combination. The keywords used were as follows: (“Simultaneous training” OR “Simultaneous exercise” OR “Concurrent training” OR “combined exercise”) AND (“Breast cancer”). The eligibility criteria were defined using the PICOS framework, which considers the population, intervention, comparison, outcomes, and study design. The inclusion and exclusion criteria are shown in Table 1.

### 2.3. Data Collection Process

Data from the included studies were independently analyzed by two reviewers (H.P.N and R.M.) and extracted into Microsoft Excel™ based on the effects of concurrent exercise in patients with breast cancer. Any discrepancies between the reviewers were resolved through discussion with a third author (D.E.).

### 2.4. Study Risk of Bias Assessment

The methodological quality of all the included studies was assessed using the Cochrane risk of bias tool [31]. The risk of bias assessment was conducted by two independent reviewers using the Cochrane risk of bias tool (Supplementary Figures S1 and S2). In case of disagreements, a third author was consulted. The terms used in classifying the studies were low risk, high risk, or unclear risk. The Review Manager software (RevMan, The Nordic Cochrane Centre, Copenhagen, Denmark) version 5.4 was used to build the risk of bias graphs.

### 2.5. Synthesis Methods and Statistical Analysis

For each study, the results before and after the intervention were analyzed according to the outcomes measured (physical, psychological, and biomarkers). The meta-analysis was analyzed according to the outcome measures for the studies with control groups (and when the data were available). In this manner, the results were synthesized using a combination of narrative synthesis and, where applicable, a meta-analysis. The choice of method was justified based on the heterogeneity of the studies included and the characteristics of their results. The results of the individual studies and syntheses were tabulated and presented visually in three main tables. Table 2 details the characteristics of the intervention (frequency, exercise, duration, and intensity), while also summarizing the outcomes measured after training and their effects. The outcomes presented in Table 3 show the percentage difference between the mean pre- and post-intervention values. This value was calculated as follows: [(mean post value ÷ mean pre-value) − 1] × 100. In Table 2, “increase” refers to a significant increase, while “decrease” refers to a significant decrease. The expression “no effect” refers to situations in which there were no significant differences in the variable. The visual presentation was structured to make it easier to understand and compare the information between the different studies included in the review. In addition, the supplementary Table S1 presents the characteristics of the population for further analysis.

The meta-analysis to evaluate the effect of concurrent training in breast cancer survivors was conducted for body mass, BMI, % fat mass, muscle strength, and VO2max using the Review Manager software (version 5.4; The Nordic Cochrane Centre, The Cochrane Collaboration, Copenhagen, Denmark). The meta-analysis was restricted to variables reported in more than four articles to ensure the robustness and reliability of the presented data [32,33]. This methodological approach was adopted to enhance the validity of the findings and to mitigate the impact of potential outliers or insufficiently supported results [32,33]. To determine the overall effect sizes, the weighted mean differences (WMD) and the standard deviations (SD) of the measures were extracted from both intervention and control groups, utilizing a random-effects model as described in the literature [34]. In cases where mean changes were not directly reported, they were calculated using the following formula: mean change = final values − baseline values. The standard deviation of the changes (SD change) was derived using the following formula: SD change = √ [(SD baseline) ^2 + (SD final) ^2 − (2R × SD baseline × SD final)], as referenced in the literature [35]. Since most studies did not report the ΔSD, a correlation coefficient (r) of 0.70 was applied [34]. The intervention effect was calculated using the standardized mean difference (SMD) for each study, along with 95% confidence intervals (95% CI). Results were deemed statistically significant at *p* ≤ 0.05. The magnitude of the intervention effect was classified according to Cohen’s categories: d values between 0.2 and 0.5 indicate a small effect size, values between 0.5 and 0.8 denote a medium effect size, and values greater than 0.8 signify a large effect size [34].

## 3. Results

### 3.1. Study Selection

**Figure 1:**
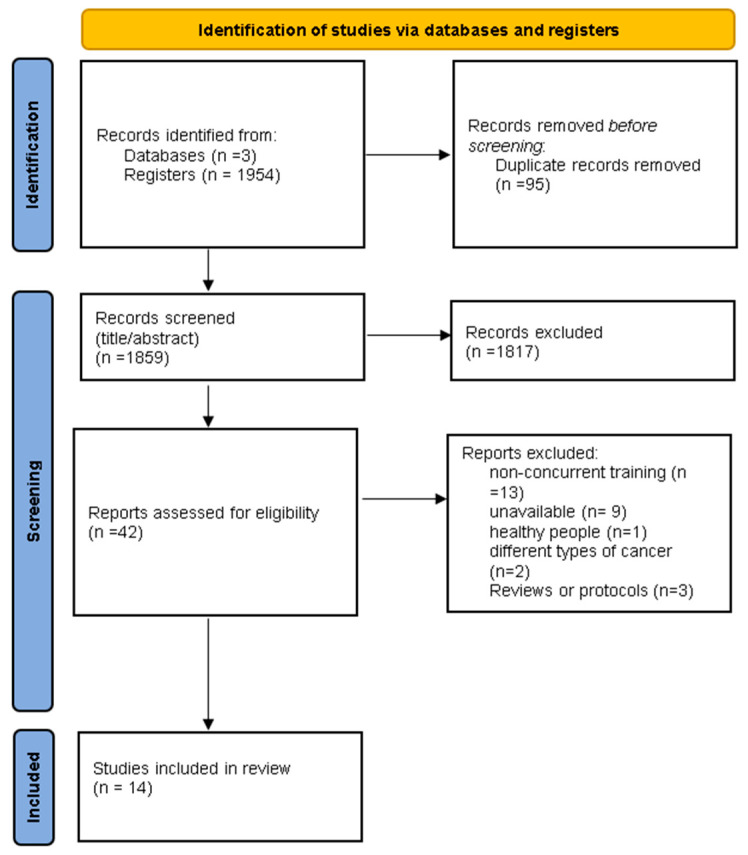
Flowchart diagram of screening studies included in this Review using the Preferred Reporting Items for Systematic Reviews and Meta-Analyses (PRISMA).

The study selection process is illustrated in Figure 1. The initial search yielded 1954 studies across the 3 databases. After the removal of duplicates, 1859 articles remained. Following a review of titles and abstracts, 1817 articles were excluded. Ultimately, 42 articles were identified as meeting the inclusion criteria based on title and abstract. After full reading, 28 articles were excluded as they did not meet the inclusion criteria. Therefore, this systematic review included a total of 14 studies comprising 1253 participants. Of these, 267 were allocated to the control group, while 503 participated in the experimental group, receiving both aerobic and resistance training (AT + RT).

### 3.2. Study Characteristics

Table 2 summarizes all outcomes and changes observed post-intervention. The characterization of the population included in the systematic reviews is shown in Supplementary Table S1 (Supplementary Table S1 provides detailed information on study populations, frequency, type, duration, and intensity). 

**Table 2:** Interventions included in each of the systematic reviews and meta-analyses.

Study	Protocol (Weeks)	Frequency	Aerobic Training			Resistance Training			
			Exercise	Duration (min)	Intensity	Exercise	Duration (min)	Set × Repetitions	Intensity
[27]	24 WKs (AT + RT)	2× WK 90 min	Cycle ergometer	20–30 min	70–80% HR	LC, LE, LP, SP, and vertical traction	40 min	2–4 × 6–10	40–60% 1RM
[29]	16 WKs (AT + RT)	2× WK 75 min	Cycle ergometer, aerobic games, treadmill running, or elliptical ergometer	35 min	7–8 Borg RPE scale	Elastic bands, DB, fit balls, suspension training, or body mass that involved the major muscle groups	40 min	2–3 × 8–12 sets with 2 min rest; exercise with 30 s rest. 1–4 WKs (2 × 12); 5–8 WKs (2 × 8); 9–12 WKs (2 × 8); 13–16 WKs (3 × 8).	6–7 RPE
[36]	12 WKs (AT + RT)	3× WK 50–60 min	Cycle ergometer treadmill, elliptical, rowing ergometer	25–30 min	55–60% VO2max; 6–12 WKs 70–75% VO2max	LE, LC, LP, CR, CP, SR, TE, and BC	30–35 min	2 × 10–12	60–75% 1RM
[37]	36 WKs (AT + RT)	3× WK 1 h 40 min	Treadmill	30 min	50–80% HR	SR, BP, LE, LP, LC, bridge, and plank	40–50 min	1–3 × 8–20 with 60–90 s rest	55–75% 1RM
[38]	16 WKs (AT + RT)	3× WK 80 min	Treadmill walking/running; rowing machine; stationary bicycle	20 min	65–80% HR_max_ (↑ every 4 WKs)	LP, CP, lunges, SR; LE; TE; LF; BC	60 min	3 × 10 (↑ every 4 WKs) without rest	80% 1RM lower body; 60% 1RM upper body
[39]	16 WKs (AT + RT)	2× WK 80 min	Treadmill walking/running, rowing machine, or cycle Ergometer	20 min	65–80% HR_max_	LP, LE, LF, CP, SR, TE, and BC	60 min	3 × 10 repetitions (↑every 4 WKs)	80% 1RM lower body; 60% 1RM upper body
[40]	8 WKs (AT + RT)	3× WK 90 min	Cycle ergometer	20–30 min	70–80% HR_peak_	CP, SP, LE, LC, LP, CR, AC, BE, BC, TE, and LPD	40–50 min	2–3 × 8–15	NS
[41]	12 WKs (AT + RT)	2× WK 60 min	Row ergometer, cycle ergometer, treadmill, walking, cross-trainer, step-up stations	15 min	Intensity was self-selected	LP, LE, LHC, BP, LPD, SR, DBSP, BC, TE, V-sit, AC, reverse AC, BPB, CPR, lunges with MB twists, WL, band shoulder IR and ER, BP, and SP	45 min	3 min per station 10–12	60% 1RM
[42]	12–18 WKs (AT + RT)	3× WK 50–60 min	NS	25–30 min	≥70% VO2max	LC, LE, CR, CP, SR, TE, and BC	30–35 min	3 × 10–12	60–75% 1RM
[43]	16 WKs (RT-HIIT)	2× WK 60 min	Cycle ergometer	NS	HIIT (3 × 3 min: 1 min rest)	The exercises targeted the main muscles groups	NS	2–3 × 8–12	70–80% 1RM
[44]	4 WKs (AT + RT)	3× WK 70 min	Cycle ergometer	30 min	60% VO2max	Squat, SP, HF, BBOR, and FP	40 min	3 × 8–12	RPE
[45]	12 WKs (AT + RT)	1× WK	Aerobic dance, yoga, bricks walking, cycling, or walking in the pool	NS	NS	CP, LPD, SR, LP, LC, and LE	NS	2–3 × 8–20	0–4 WKs (7–12 RPE); 5–12 WKs (10–15 RPE)
[46]	12 WKs (AT + RT)	3× WK 60 min	Cycle ergometer	30 min	50–90% HR	HF, HE, SP, Swiss ball squatting, FP, and curved paddling	30 min	3 × 12 Sets 1 min rest	Body mass, 1 kg DB and band (moderate intensity) 5th WK ↑ 1 kg
[47]	12 WKs (AT + RT + F)	3/5× WK 60 min	Cycle ergometer	30 min	50–90% HR	HF, HE, SP, squatting with Swiss ball, FP, and lifting exercises for the dorsal muscles	30 min	3 × 12 Sets 1 min rest	Body mass, 1 kg DB and band (moderate intensity) setting; 5–8 WKs ↑ 1 kg and band at high setting.

Abbreviations: ↑ = increase; AC = abdominal crunch; AT = aerobic training; BBOR = barbell bent over row; BC = bicep curl; BE = back extension; BMI = body mass index; BP = bench press; BPB = band pulldown with bridge; CPR = Cuban press raise; CR = calf raise; DB = dumbbell; DBP = diastolic blood pressure; DBSP = dumbbells shoulder press; ER = external rotation; F = flexibility; FP = French press; HF = hip flexion; HIIT = high-intensity interval training; HE = hip extension; HRpeak = peak heart rate; IR = internal rotation; kg = kilogram; LC = leg curl; LE = leg extension; LF = leg flexion; LHC = lying hamstring curl; LP = leg press; LPD = Lat pulldown; MB = medicine ball; min = minute; NS = not specified; RPE = Borg’s rating of perceived exertion; RT = resistance training; S = seconds; SP = shoulder press; SR = seated row; TE = tricep extension; VO2max = maximal oxygen consumption; WK = week; WL = walking lunge.

The included studies were conducted in various countries, including Canada [36,42], Italy [27], Brazil [37,44,46,47], Japan [45], Spain [29,40], New Zealand [41], USA [38,39], and Sweden [43]. The study population consisted of breast cancer survivors, with varying age ranges specified in six studies (two aged 18 or older [36,42]; one aged 50 years and over [37]; one aged between 40 and 60 years [40]; two aged between 30 and 59 years [44,46]; one aged between 20 and 75 years [45]; one aged between 18 and 65 years [29]; one aged between 18 and 70 years [43]; and one aged between 40 and 70 years [41]). Seven articles included patients in stages I–III [36,37,38,39,42,43,44], with one in stages I–II [40], while others did not specify disease stages [27,29,41,45,46,47].

The intervention period also varied across the included studies: three were during treatment [44,46,47], two were between 2 and 5 years post-treatment [29,40], three were initiating chemotherapy [36,42,43], two had completed all treatments over six months ago [38,39], one completed adjuvant treatment at least 8 weeks ago [41], and one involved postmenopausal patients undergoing aromatase inhibitor therapy [37]. 

### 3.3. Risk of Bias in Studies

The generation of random sequences was identified at approximately 65%. Allocation concealment was reported to be around 85%. Blinding of outcome assessment achieved a rate of 75%. There was a low risk of bias (100%) for various issues, such as incomplete outcome data, selective reporting, and other biases. However, blinding of participants and personnel was the item with the lowest implementation, standing at about 60%. A risk of bias graph and summary can be found in the Supplementary Files (Figures S1 and S2).

### 3.4. Intervention

The interventions for concurrent training in breast cancer survivors varied from 4 weeks to 36 weeks, with training frequencies ranging from 1 to 3 sessions per week. These interventions were implemented at various stages of treatment or disease progression. Upon reviewing the selected articles, there was substantial diversity in the types of aerobic training equipment and methods used, including cycle ergometers, treadmills, ellipticals, rowing ergometers, aerobic dance, yoga, brisk walking, walking, pool walking, and aerobic games, with sessions typically lasting between 20 and 35 min.

The methods used to determine exercise intensities varied among the articles, with some using 60% of maximal oxygen consumption (VO2max), and others using heart rate (HR) targets ranging from 50% to 80%, VO2 peak targets (55–75%), and Borg’s rating of perceived exertion (RPE) scale. Regarding resistance training, sessions typically included 1 to 4 sets of 6 to 20 repetitions, lasting between 30 and 60 min. The methods used to calculate training intensity varied across studies, with two studies not specifying intensity, three using the one-repetition maximum (1RM) method with intensities ranging from 40% to 80%, and others relying on Borg’s RPE scale. All articles specified the types of resistance exercises employed, which are detailed in Table 2 for further analysis.

### 3.5. Outcomes

The outcomes analyzed in the selected studies can be categorized into physical, psychological, and biomarker variables. According to the reported results, all interventions involving concurrent training demonstrated positive effects on cardiorespiratory fitness, particularly VO2max [25,31,33,35,36,39]. Ten studies highlighted changes in physical parameters, including improvements in body composition [25,27,31,32,33,34,35,38,39,40], upper and lower limb strength [25,27,31,32,36,39,40], and flexibility [36].

With respect to psychological outcomes, several studies noted positive changes, including enhanced quality of life [40], reduced fatigue [40], and improved sleep quality [37]. In terms of biomarker outcomes, only four of the fourteen studies [27,34,38,39] reported positive changes, specifically in blood pressure [34,38], insulin resistance [38], proinflammatory markers [38], estradiol levels [38], and neutrophil-to-lymphocyte ratio [27]. However, it should be noted that while these improvements were observed, not all were statistically significant, as shown in Table 3.

**Table 3:** Intervention group: outcomes measured after the training period.

Outcome Measure	Studies	Effect Post-Training		
		Increase	Decrease	No Effect
Biomarkers				
Hemoglobin (g·dL^−1^)	[40]			[40] (0.7%)
Hematocrit (%)	[40]			[40] (0.5%)
Erythrocyte count (×10^6^·mm^3^)	[40]			[40] (2.2%)
Systolic blood pressure (mmHg)	[39,41,44,45]		[39] (9.6%)	[41] (2.4%)
			[45] (4.7%)	[44] (6.1%)
Diastolic blood pressure (mmHg)	[39,41,44,45]		[39] (13.3%)	[41] (2.5%)
			[45] (1.7%)	[44] (9.4%)
Pulse wave velocity	[41]		[41] (4.3%)	
HDL-C (mg/dL)	[39]	[39] (40.1%)		
Triglycerides (mg/dL)	[39]		[39] (48.2%)	
Glucose (mg/dL)	[39]		[39] (16.4%)	
Metabolic syndrome z-score	[39]		[39] (53.1%)	
ATP III score	[39]		[39] (120%)	
Insulin (μU/mL)	[39]		[39] (47.7%)	
HOMA-IR	[39]		[39] (48.0%)	
IGF-1 (ng/mL)	[39]		[39] (10.7%)	
IGFBP-3 (ng/mL)	[39]	[39] (15.4%)		
hs-CRP (ng/mL)	[39]		[39] (28.6%)	
Leptin (ng/mL)	[39]		[39] (28.1%)	
Adiponectin (μg/mg)	[39]	[39] (43.2%)		
Total cholesterol (mg/dL)	[39]		[39] (22.0%)	
IL-6 (pg/mL)	[39]		[39] (53.9%)	
IL-8 (pg/mL)	[39]		[39] (39.1%)	
TNF-α (pg/mg)	[39]		[39] (66.6%)	
SHBG (nmol/L)	[39]	[39] (15.0%)		
Estradiol (pg/mL)	[39]		[39] (26.1%)	
Free testosterone (pg/mL)	[39]			[39] (24.6%)
Pulse wave velocity (m/s)	[41]			[41] (3.2%)
Augmentation pressure (mmHg)	[41]			[41] (9.4%)
Augmentation index (%)	[41]			[41] (7.0%)
Central systolic blood pressure (mmHg)	[41]			[41] (2.6%)
Central pulse pressure (mmHg)	[41]			[41] (2.5%)
Heart rate (bpm)	[41]			[41] (1.3%)
Rate pressure product (mmHg/bpm)	[41]			[41] (1.4%)
Peripheral pulse pressure (mmHg)	[41]			[41] (3.9%)
Mean arterial pressure (mmHg)	[41]			[41] (2.2%)
Neutrophil-to-lymphocyte ratio	[29]		[29] (22.2%)	
Cardiorespiratory fitness				
VO2max (mL·kg^−1^·min^−1^)	[27,37,41,44] [46,47]	[27] (32.4%)		
		[37,41] (14.7%)		
		[44] (17.1%)		
		[46] (20.2%)		
		[47] (20.41%)		
VO2peak (mL·kg^−1^·min^−1^)	[40,43,45]	[40] (8.3%)		
		[43] (8.1%)		
		[45] (12.9%)		
VO2peak (mL·min^−1^)	[43]	[43] (11%)		
PPO: Body mass (w·kg^−1^)	[40]	[40] (23.53%)		
HR_peak_ (b·min^−1^)	[40]			[40] (0.6%)
RER_peak_	[40]			[40] (8.7%)
Ventilatory Threshold				
VO2peak (mL·kg^−1^·min^−1^) at VT	[40]			[40] (4.4%)
PPO: body mass (w·kg^−1^) at VT	[40]			[40] (25%)
Heart rate (b·min^−1^)	[40]			[40] (1.7%)
Respiratory exchange ratio at VT	[40]			[40] (0%)
Distance (m)	[41]	[41] (22.2%)		
Rating of perceived exertion (OMNI)	[41]			[41] (12.0%)
Cardiorespiratory fitness (MET)	[29]			[29] (7.1%)
Moderate-vigorous physical activity (min/week)	[29]			[29] (2.4%)
Muscle strength				
Bench press (n°Rep)	[40]	[40] (92.3%)		
Leg press (n°Rep)	[40]	[40] (68.2%)		
Leg press (kg)	[27,37]	[27] (28.8%)		
		[37]		
Leg extension (kg)	[27]	[27] (18.5%)		
Leg curl (kg)	[27]	[27] (12.5%)		
Shoulder press (kg)	[27]	[27] (46.6%)		
Vertical traction (kg)	[27]	[27] (18.6%)		
Bench 1RM (kg)	[37,41]	[37]		
		[41] (17.1%)		
Knee extension (Nm)	[41]	[41] (3.3%)		
Knee flexion (Nm)	[41]			[41] (18.4%)
Static strength (kgf)—right arm	[46,47]	[46] (23.9%)		
		[47] (23.2%)		
Static strength (kgf)—left arm	[46,47]	[47] (15.6%)		[46] (14.7%)
Isometric mig-thigh pull (kg)	[43]	[43] (13.9%)		
Handgrip surgery side (kg)	[43]	[43] (3.6%)		
Handgrip non-surgery side (kg)	[43]	[43] (2.4%)		
Handgrip (kg)	[29]			[29] (0%)
Lower limb functionality				
Sit and stand test (s)	[29,40]	[40] (9.0%)	[29] (27.0%)	
Flexibility (cm)	[47]	[47] (37.46%)		
Body Composition				
Body mass (kg)	[29,37,39,40,41,43,44,45,46]	[40] (0.6%)	[39] (4.8%) [45] (9.6%)	[29] (0.9%) [37] (2.3%) [41] (0%) [43] (1.2%) [44] (9.5%)[45] (0%) [46] (1.2%)
Free fat mass (kg)	[27,29,37,39,40,41,46]	[37] (1.8%)		[29] (1.8%) [46] (2.6%) [41] (0.4%)
		[40] (4.7%)		
		[39] (5.2%)		
Body cell mass (%)	[27]			[27] (5.7%)
Fat mass (kg)	[37,39,40,41]		[40] (9.6%) [39] (18.6%)	[37] (6.6%) [41] (0.7%)
Fat mass (%)	[27,29,37,39,40,41,46]		[27] (6.4%) [29] (2.4%) [39] (10.5%) [40] (9.6%) [46] (3.9%)	[37] (4.9%) [41] (1.4%)
Trunk fat mass (kg)	[37,39]		[39] (17.9%)	[37] (5.4%)
Waist circumference (cm)	[29,39,46]		[29] (2.1%) [39] (7.6%)	[46] (1.9%)
Hip circumference (cm)	[39,46]		[39] (7.4%)	[46] (0.6%)
Abdominal circumference (cm)	[45,46]		[45] (7.9%)	[46] (1.3%)
Circumference of the right thigh (cm)	[46]			[46] (4.55%)
Circumference of the left thigh (cm)	[46]			[46] (3.2%)
BMI (kg/m^2^)	[29,39,41,43,44,45,46,47]		[39] (4.9%) [45] (9.0%)	[29] (2.8%) [41] (0.4%) [43] (1.2%) [44] (3.9%) [46] (0%) [47] (0.5%)
Appendicular skeletal muscle index (kg/m^2^)	[39]	[39] (27.6%)		
Waist–hip ratio (cm/cm)	[46]			[46] (0%)
Conicity index (m/kg/m)	[46]			[46] (0%)
Reciprocal ponderal index (cm/kg)	[46]			[46] (0%)
Bone mineral density (g.cm^−2^)	[29]			[29] (0%)
FACIT-F				
ACIT-F trial outcome	[27]	[27] (12.0%)		
FACT-G	[27]	[27] (16.4%)		
FACIT-F	[27]	[27] (13.7%)		
Body Image				
Body Stigma	[46]			[46] (1.8%)
Limitations	[46]		[46] (37.4%)	
Concerns about the arm	[46]			[46] (11.9%)
Body concerns	[46]			[46] (33.9%)
Transparency	[46]			[46] (19.4%)
Vulnerability	[46]			[46] (22.0%)
PFS Questionnaire				
Behavior/daily life CRF	[43]		[43] (2.9%)	
Emotional/affective CRF	[43]		[43] (5.1%)	
Cognitive CRF	[43]			[43] (1.1%)
Sensory/physical	[43]			[43] (0.9%)
Total CRF	[43]		[43] (9.9%)	
EORTC-QLQ_C30 questionnaire				
Global/quality of life	[43]			[43] (0.5%)
Physical functioning	[43]			[43] (3.9%)
Emotional functioning	[43]			[43] (6.4%)
Role functioning	[43]			[43] (16.7%)
Cognitive functioning	[43]			[43] (1.4%)
Social functioning	[43]			[43] (5.1%)
Fatigue	[43]			[43] (5.6%)
Nausea and vomiting	[43]			[43] (43.5%)
Pain	[43]			[43] (1.4%)
Dyspnea	[43]			[43] (33.7%)
Insomnia	[43]			[43] (16.6%)
Appetite loss	[43]			[43] (36.1%)
Constipation	[43]			[43] (66.9%)
Diarrhea	[43]			[43] (48.9%)
Financial difficulties	[43]			[43] (13.9%)
Fatigue (PERFORM questionnaire score)	[27]		[27] (46.9%)	
Revised Piper fatigue scale (PFS-R)	[47]			[47] (36.1%)
Pittsburgh Sleep Quality Index (PSQI)				
Global sleep quality	[37]		[37] (40.9%)	
Subjective sleep quality	[37]		[37] (64.8%)	
Sleep latency	[37]		[37] (53.5%)	
Sleep duration	[37]		[37] (25.1%)	
Sleep disturbance	[37]		[37] (42.1%)	
Sleep medication	[37]		[37] (49.3%)	
Daytime dysfunction	[37]		[37] (38.1%)	
Poor sleepers (%)	[37]		[37] (67.3%)	
MSAS questionnaire				
Symptom burden	[43]			[43] (16.7%)
Physical symptoms	[43]		[43] (8.5%)	
Psychological symptoms	[43]			[43] (5.0%)
Total symptoms	[43]		[43] (24.2%)	

Abbreviations: BMI = body mass index, CRF = Cancer-related fatigue, FACIT-F = functional assessment of chronic illness therapy—fatigue, FACT-G = functional assessment of cancer therapy—general, HDL-C = high-density lipoprotein-cholesterol, HOMA-IR = homeostasis model assessment of insulin resistance, HR_peak_ = peak heart rate, hs-CRP = high-sensitivity C-reactive protein, IGF-1 = insulin-like growth factor-1, IGFBP-3 = insulin-like growth factor-binding protein-3, IL = interleukin, RER_peak_ = peak respiratory exchange ratio, RM = repetition maximum, SHBG = sex hormone-binding globulin, TNF-α = tumor necrosis factor-α, VO2max = maximal volume of oxygen consumed per minute, VT = ventilatory threshold.

### 3.6. Meta-Analysis

**Figure 2:**
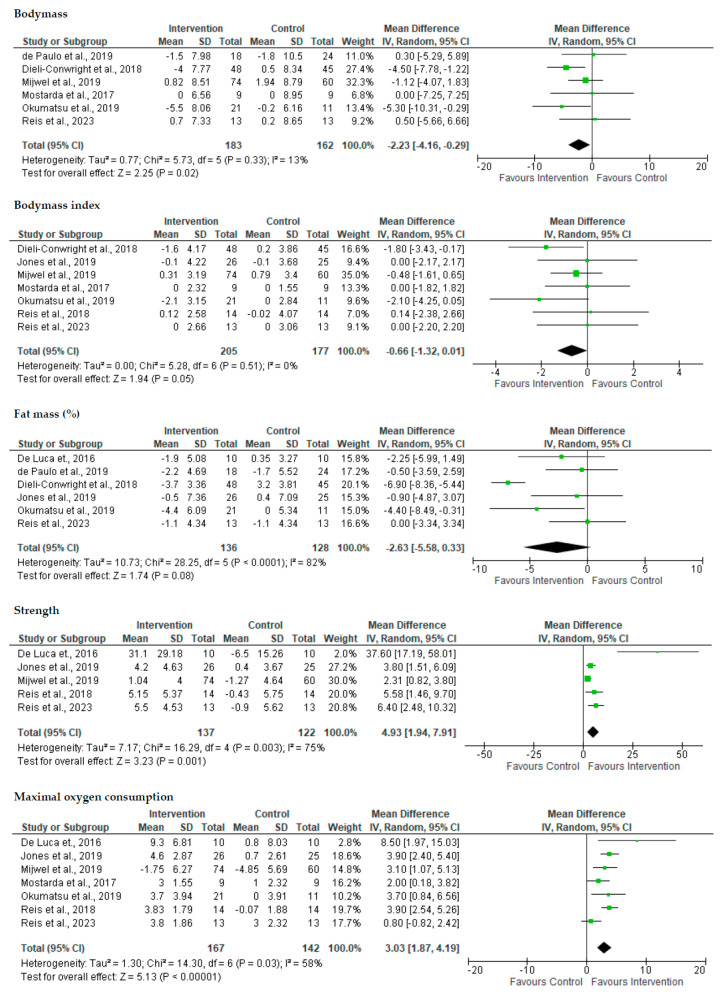
Forest plot of the meta-analysis of the effect of concurrent training in breast cancer survivors [27,37,39,41,43,44,45,46,47].

The meta-analysis results for body composition and muscle strength are shown in Figure 2. There were significant differences in the overall effect size for body mass (*p* = 0.02) and strength (*p* < 0.001), while for BMI (*p* = 0.05) and % fat mass (*p* = 0.08), no differences were observed. For muscular strength, assessments of all muscular regions were included (e.g., 1RM leg press/bench press/handgrip and isometric mig-thigh pull).

The results of the meta-analysis for the cardiorespiratory parameters are shown in Figure 2. The variable that was considered for analysis was the VO2max, and significant differences were found (*p* ≤ 0.01). It is interesting to note that, contrary to what was mentioned in the body composition parameter, only one of the articles presented does not show significant differences, even though it is right at the limit.

## 4. Discussion

This systematic review and meta-analysis aimed to synthesize the scientific evidence on the impact of concurrent training on physiological, psychological, and social outcomes, as well as potential adverse effects in breast cancer survivors. Specifically, it sought to identify the physiological and psychological effects, as well as major biomarkers, of concurrent training in this population. The main findings of this review were as follows: (i) a decrease in body mass; (ii) a decrease in BMI; (iii) an increase in muscle strength; (iv) an increase in VO2max. The review encompasses 14 articles that exclusively studied breast cancer patients, involving adult populations with varying age ranges (18–75 years). The included studies covered patients from stage I to III of the disease. The outcomes analyzed were categorized into three main parameters: physical, psychological, and biomarkers.

### 4.1. Effect of Concurrent Training on Body Composition

During breast cancer treatment, patients often experience significant changes in body composition. Numerous scientific studies have examined these changes, primarily focusing on weight gain, alterations in muscle mass, and fat distribution [48]. These changes in body composition can have several negative consequences, including decreased quality of life and self-esteem, as well as an increased risk of associated diseases, such as diabetes, cardiovascular diseases, and cancer recurrence [48,49]. Excess fat, particularly visceral fat, is known to adversely affect the hormonal and metabolic systems, increasing insulin levels and insulin resistance, which are associated with poorer outcomes in the diagnosis and treatment of breast cancer [24]. Regarding body composition, our meta-analyses revealed significant differences, specifically in body mass. In contrast, there were no significant differences in BMI, free fat mass, and % fat mass. The response of free fat mass appears to vary based on the intensity of training across studies [29,37,39,41,46]. Studies prescribing intensities between 55% and 80% of 1RM showed significant increases in free fat mass (kg) [37,39], suggesting that moderate to high intensities effectively promote muscle hypertrophy [50].

Conversely, studies where training intensity was either unspecified [46] or determined using the Borg scale [29] reported no significant effects on fat-free mass, emphasizing the critical need for precise quantification of strength training intensity [51,52]. Notably, in one study where %1RM was quantified [41], no significant changes in fat-free mass were observed. Regarding fat mass, no significant differences were detected, potentially due to the circuit nature of concurrent training, which often includes shorter rest periods and may not provide a sufficient progressive overload to effectively increase fat-free mass or reduce fat mass [53].

### 4.2. Effect of Concurrent Training on Muscle Strength

Additionally, muscle dysfunction and sarcopenia are prevalent among breast cancer patients, contributing to poor performance status, increased mortality risk, and heightened side effects from cancer treatments [54]. Engaging in exercise, particularly resistance training, has been shown to mitigate these issues by improving muscle strength and function [54]. Enhanced muscle mass and strength help counteract the adverse effects of chemotherapy and other treatments, ultimately improving survival rates and quality of life for breast cancer survivors [54]. Therefore, incorporating resistance training during and after cancer treatment is essential for maintaining and improving musculoskeletal health, which is closely linked to better treatment outcomes and recovery [54].

Meta-analyses revealed significant improvements in muscle strength across most assessed variables. However, knee flexion (Nm) [41], handgrip strength (kg) [29], and static strength (kgf) of the left arm [46] did not show significant improvements within the experimental group, although they did improve compared to the control group. These results suggest that, while the training was effective relative to no training, it may not have been sufficiently intense or specific to elicit significant enhancements in these particular strength measures within the experimental group. Additionally, the sit and stand test [29] demonstrated discrepancies, likely due to variations in the specification of intensity (%1RM) across different studies [29,46].

The use of the Borg scale to determine exercise intensity in some studies [29,44] may not be optimal for this population. Perceived exertion can vary widely among individuals, leading to inconsistent training intensities and potentially lower effort than intended [51,55,56]. In untrained women, self-selected or perceived exertion often results in lower intensities, possibly due to concerns about injury or excessive muscle gain [56].

### 4.3. Effect of Concurrent Training on Cardiorespiratory Fitness

Regarding cardiorespiratory fitness, VO2max demonstrated significant improvements across studies, regardless of training duration, frequency, or intensity [27,37,40,41,43,44,45,46,47]. These results are consistent with the findings of the meta-analysis and align with existing research, which indicates that concurrent training enhances the cardiorespiratory capacity of breast cancer survivors, with some studies reporting increases of up to 16% [41,57].

Improving cardiorespiratory fitness in cancer survivors is essential for combating fatigue and mitigating the adverse side effects of chemotherapy [58]. Chemotherapy, while effective for cancer treatment, often induces debilitating symptoms, such as fatigue, weakness, and cardiovascular decline [10,11,12]. By improving cardiorespiratory fitness through concurrent training, survivors can enhance their physical capacity, endurance, and oxygen utilization efficiency. This improved fitness directly supports the body’s ability to cope with chemotherapy-induced fatigue [59], helping to maintain energy levels, reduce feelings of exhaustion, and optimize physiological functioning during and after treatment [60]. Furthermore, enhanced cardiovascular health may contribute to better tolerance of chemotherapy sessions, potentially reducing treatment interruptions and improving overall treatment adherence [59].

### 4.4. Effect of Concurrent Training on Psychological Outcomes

In terms of quality of life, no significant differences were found when comparing concurrent training with the control group [43]. However, aerobic training alone did show differences in specific quality of life parameters, such as emotional functioning, role functioning, fatigue, appetite loss, and constipation [43]. These findings align with the literature suggesting that aerobic exercise can enhance self-efficacy and certain psychological aspects, contributing to improved emotional and social quality of life outcomes [61].

Variations were observed in physical and total symptoms as assessed by the Memorial Symptom Assessment Scale [43]. Aerobic training also demonstrated improvements in symptom burden and psychological variables compared to concurrent training [43], possibly due to the psychological benefits and enhanced self-efficacy associated with aerobic exercise.

The findings related to the Physical Functioning Scale questionnaire showed significant differences across nearly all variables, except for sensory/physical and cognitive aspects [43]. This indicates that AT-HIIT training may have a more direct impact on quality of life and fatigue, potentially through enhanced cerebral oxygenation [43]. Moreover, concurrent training led to decreased Pittsburgh Sleep Quality Index (PSQI) scores, suggesting an improvement in sleep quality [38], consistent with findings in other studies [62,63].

### 4.5. Effect of Concurrent Training on Biomarkers

The analysis of biomarkers plays a crucial role in both diagnosing and treating breast cancer. For instance, elevated levels of testosterone and CRP (C-reactive protein) are linked to increased breast cancer risk, whereas higher levels of SHBG (sex hormone-binding globulin) are associated with reduced risk [17]. Integrating measurements of testosterone, SHBG, and CRP into diagnostic models could enhance breast cancer detection, particularly among individuals of normal weight [17]. Conversely, obesity is linked to insulin resistance and dysmetabolism, including hyperinsulinemia and increased production of IGF-I [18]. Hyperinsulinemia stimulates ovarian androgen synthesis while inhibiting SHBG production, thereby elevating testosterone levels [18]. This enhances estradiol bioavailability and promotes insulin resistance, thereby increasing breast cancer risk [18].

Our findings indicate significant increases in HDL-C (mg/dL), IGFBP-3 (mg/mL), adiponectin (μg/mg), and SHBG (nmol/L) after 12 weeks of competitive training. These improvements in lipid profile and metabolic markers are beneficial for cardiovascular and metabolic health, potentially preventing breast cancer onset or progression [18,19,64]. Additionally, other metabolic and inflammatory variables (e.g., IGF-1, IL-6, IL-8, TNF-α, HOMA-IR, estradiol, and hs-CRP) decreased with concurrent training, suggesting an anti-inflammatory effect and improved insulin sensitivity. These results are consistent with randomized clinical trials that investigated changes in insulin, IGF axis, adiponectin, IL-6, TNF-α, and CRP following exercise interventions in breast cancer patients [65,66].

However, our study found that free testosterone (pg/mL) did not change with concurrent training [39]. Systematic reviews have shown that higher testosterone levels correlate with breast cancer recurrence, whereas lower levels are associated with better disease-free survival [18,56]. These results suggest that competitive training might be a beneficial strategy for modulating hormone levels and maintaining a healthy weight, crucial for reducing breast cancer risk and improving prognosis [17,18,64].

Furthermore, physical exercise’s effect on tumor-infiltrating immune cells and mediators relevant to the tumor microenvironment, such as cytokines, represents an important mechanism for enhancing cancer prognosis [66]. Nevertheless, it is essential to acknowledge that while our findings align with the existing literature, these biomarkers were only assessed in the context of concurrent training.

Regarding blood pressure, the results across studies [39,41,44,45] demonstrated inconsistencies. Specifically, two studies reported no significant changes: one that utilized self-selected intensity for aerobic exercise [41] and another with a brief 4-week training duration [44]. Additionally, for blood pressure-related variables, such as pulse wave velocity (m/s), augmentation pressure (mmHg), augmentation index (%), central systolic blood pressure (mmHg), central pulse pressure (mmHg), heart rate (bpm), rate pressure product (mmHg/bpm), peripheral pulse pressure (mmHg), and mean arterial pressure (mmHg), the study that employed self-selected intensity [44] also did not observe significant changes.

Self-selected intensity may result in insufficient cardiovascular stress [56], thereby limiting the capacity to induce significant cardiovascular adaptations. Similarly, the short duration of the intervention in one study [44] may not have been long enough to elicit substantial alterations in blood pressure metrics [67,68].

### 4.6. Recommendations and Future Directions

This systematic review and meta-analysis present several limitations. Firstly, not all articles provided detailed information on the intensities, volumes, and specific exercises performed during the interventions. Secondly, there was variability in intervention duration and the stage of disease among participants. Thirdly, our study acknowledged that the differences between pre- and post-exercise moments, regardless of the treatment phase, represent a limitation. Future studies should consider the following recommendations to enhance the reliability and applicability of findings: conduct long-term studies to evaluate the sustained effects of concurrent training in breast cancer survivors; investigate the underlying mechanisms of physiological adaptations to concurrent training in this population; and explore how exercise impacts tumor immune cells and inflammatory mediators, offering valuable insights. Additionally, future research should encompass diverse populations, taking into account multiple variables, such as age and cancer stage.

## 5. Conclusions

This systematic review and meta-analysis demonstrated that concurrent training, incorporating both strength and aerobic exercises, can yield substantial enhancements in decreasing body mass, increasing muscle strength, cardiorespiratory fitness, and improving various metabolic and inflammatory markers. Moreover, concurrent training seems to positively impact quality of life, sleep quality, and fatigue levels in breast cancer survivors. However, the effectiveness of such training hinges on various factors, like exercise intensity, duration, frequency, and the tailored nature of training protocols. Specifically, accurately defining exercise intensity (e.g., using %1RM rather than the Borg scale) appears pivotal in achieving meaningful improvements across health parameters. Programs with a shorter duration or self-selected intensities may not suffice to produce significant changes, particularly in cardiovascular and metabolic health metrics. In conclusion, this review underscores the importance of the precise prescription of exercise intensity and duration to maximize the benefits of concurrent training for breast cancer survivors. Tailoring training programs focusing on clearly defined intensity levels is essential for substantially improving diverse health outcomes.

## Figures and Tables

**Table 1:** Eligibility criteria following the PICOS (population, intervention, comparison, outcomes, and study design) approach.

Category	Inclusion Criteria	Exclusion Criteria
Population	Individuals diagnosed with breast cancer at any stage of treatment (awaiting, in progress, or have completed any form of cancer treatment).	Children and adolescents (<18 years).
Intervention	Concurrent training during the same training session.	Online studies and articles where the effect of concurrent training did not occur within the same training session.
Comparison	Intra-group comparison (pre- and post-test of the group undergoing concurrent training) and inter-group comparison (post-tests between the group undergoing the post-training period after intervention and after placebo of the control group).	Lack of an intervention group for comparison.
Outcomes	Changes from pre-test to post-test in cardiorespiratory fitness (i.e., VO2max, VO2peak), or changes from pre-test to post-test in strength (i.e., 1RM or × RM tests), functional capacity (i.e., sit and stand tests), flexibility, balance, psychological assessments (i.e., quality of life, fatigue), or biomarkers (i.e., insulin resistance, inflammatory markers, estradiol levels).	No pre-test or post-test data.
Study design	Randomized and non-randomized controlled trials.	Systematic review and meta-analysis articles.

Abbreviations: RM = repetition maximum; VO2max = maximal oxygen consumption; VO2peak = peak oxygen uptake.

## Data Availability

The data that support the findings of this study are available from the corresponding author upon reasonable request due to ethical reasons.

## Supplementary Materials

The following supporting information can be downloaded at: https://www.mdpi.com/article/10.3390/healthcare13010033/s1, Figure S1: Judgments about each risk-of-bias item for each included study: + indicates low risk, ? indicates unclear risk, − indicates high risk; Figure S2: Risk of bias item presented as percentages across all included studies; Table S1: Characterization of the population included in the systematic reviews included.

## References

[1] Sung H., Ferlay J., Siegel R.L., Laversanne M., Soerjomataram I., Jemal A., Bray F. (2021). Global Cancer Statistics 2020: GLOBOCAN Estimates of Incidence and Mortality Worldwide for 36 Cancers in 185 Countries. CA A Cancer J. Clin..

[2] Courneya K.S., Karvinen K.H. (2007). Exercise, aging, and cancer. Appl. Physiol. Nutr. Metab. Physiol. Appl. Nutr. Et Metab..

[3] Antunes P., Esteves D., Nunes C., Joaquim A., Pimentel F.L., Fonseca-Moutinho J. (2019). Health-related quality of life and physical fitness in breast cancer patients: The impact of a supervised physical exercise program in women with no exercise experience. Psychol. Health Med..

[4] Iwase T., Wang X., Shrimanker T.V., Kolonin M.G., Ueno N. (2021). T Body composition and breast cancer risk and treatment: Mechanisms and impact. Breast Cancer Res. Treat..

[5] McNeely M.L., Campbell K.L., Rowe B.H., Klassen T.P., Mackey J.R., Courneya K.S. (2006). Effects of exercise on breast cancer patients and survivors: A systematic review and meta-analysis. CMAJ Can. Med. Assoc. J. J. De L’association Medicale Can..

[6] McTiernan A., Friedenreich C.M., Katzmarzyk P.T., Powell K.E., Macko R., Buchner D., Pescatello L.S., Bloodgood B., Tennant B., Vaux-Bjerke A. (2019). Physical Activity in Cancer Prevention and Survival: A Systematic Review. Med. Sci. Sports Exerc..

[7] Spei M.E., Samoli E., Bravi F., La Vecchia C., Bamia C., Benetou V. (2019). Physical activity in breast cancer survivors: A systematic review and meta-analysis on overall and breast cancer survival. Breast.

[8] Torre L.A., Siegel R.L., Ward E.M., Jemal A. (2016). Global Cancer Incidence and Mortality Rates and Trends-An Update. Cancer Epidemiol. Biomark. Prev. A Publ. Am. Assoc. Cancer Res. Cosponsored By Am. Soc. Prev. Oncol..

[9] Cheema B.S., Kilbreath S.L., Fahey P.P., Delaney G.P., Atlantis E. (2014). Safety and efficacy of progressive resistance training in breast cancer: A systematic review and meta-analysis. Breast Cancer Res. Treat..

[10] Champ C.E., Carpenter D.J., Diaz A.K., Rosenberg J., Ackerson B.G., Hyde P.N. (2023). Resistance Training for Patients with Cancer: A Conceptual Framework for Maximizing Strength, Power, Functional Mobility, and Body Composition to Optimize Health and Outcomes. Sports Med..

[11] De Backer I.C., Van Breda E., Vreugdenhil A., Nijziel M.R., Kester A.D., Schep G. (2007). High-intensity strength training improves quality of life in cancer survivors. Acta Oncol..

[12] Soriano-Maldonado A., Carrera-Ruiz Á., Díez-Fernández D.M., Esteban-Simón A., Maldonado-Quesada M., Moreno-Poza N., García-Martínez M.D.M., Alcaraz-García C., Vázquez-Sousa R., Moreno-Martos H. (2019). Effects of a 12-week resistance and aerobic exercise program on muscular strength and quality of life in breast cancer survivors: Study protocol for the EFICAN randomized controlled trial. Medicine.

[13] de Jesus Leite M.A.F., Puga G.M., Arantes F.J., Oliveira C.J.F., Cunha L.M., Bortolini M.J.S., Penha-Silva N. (2018). Effects of combined and resistance training on the inflammatory profile in breast cancer survivors: A systematic review. Complement. Ther. Med..

[14] Hayes S.C., Newton R.U., Spence R.R., Galvão D.A. (2019). The Exercise and Sports Science Australia position statement: Exercise medicine in cancer management. J. Sci. Med. Sport.

[15] Meneses-Echávez J.F., González-Jiménez E., Ramírez-Vélez R. (2015). Effects of supervised exercise on cancer-related fatigue in breast cancer survivors: A systematic review and meta-analysis. BMC Cancer.

[16] Peel A.B., Thomas S.M., Dittus K., Jones L.W., Lakoski S.G. (2014). Cardiorespiratory fitness in breast cancer patients: A call for normative values. J. Am. Heart Assoc..

[17] Arthur R.S., Dannenberg A.J., Rohan T.E. (2021). The association of prediagnostic circulating levels of cardiometabolic markers, testosterone and sex hormone-binding globulin with risk of breast cancer among normal weight postmenopausal women in the UK Biobank. Int. J. Cancer.

[18] Venturelli E., Orenti A., Fabricio A.S.C., Garrone G., Agresti R., Paolini B., Bonini C., Gion M., Berrino F., Desmedt C. (2018). Observational study on the prognostic value of testosterone and adiposity in postmenopausal estrogen receptor positive breast cancer patients. BMC Cancer.

[19] Dieli-Conwright C.M., Orozco B.Z. (2015). Exercise after breast cancer treatment: Current perspectives. Breast Cancer (Dove Med. Press).

[20] Holmes M.D., Chen W.Y., Feskanich D., Kroenke C.H., Colditz G.A. (2005). Physical activity and survival after breast cancer diagnosis. JAMA.

[21] Courneya K.S., Segal R.J., Mackey J.R., Gelmon K., Reid R.D., Friedenreich C.M., Ladha A.B., Proulx C., Vallance J.K., Lane K. (2007). Effects of aerobic and resistance exercise in breast cancer patients receiving adjuvant chemotherapy: A multicenter randomized controlled trial. J. Clin. Oncol. Off. J. Am. Soc. Clin. Oncol..

[22] De Backer I.C., Schep G., Backx F.J., Vreugdenhil G., Kuipers H. (2009). Resistance training in cancer survivors: A systematic review. Int. J. Sports Med..

[23] Ficarra S., Thomas E., Bianco A., Gentile A., Thaller P., Grassadonio F., Papakonstantinou S., Schulz T., Olson N., Martin A. (2022). Impact of exercise interventions on physical fitness in breast cancer patients and survivors: A systematic review. Breast Cancer.

[24] Picon-Ruiz M., Morata-Tarifa C., Valle-Goffin J.J., Friedman E.R., Slingerland J.M. (2017). Obesity and adverse breast cancer risk and outcome: Mechanistic insights and strategies for intervention. CA Cancer J. Clin..

[25] Montaño-Rojas L.S., Romero-Pérez E.M., Medina-Pérez C., Reguera-García M.M., de Paz J.A. (2020). Resistance Training in Breast Cancer Survivors: A Systematic Review of Exercise Programs. Int. J. Environ. Res. Public Health.

[26] García-Pallarés J., Izquierdo M. (2011). Strategies to optimize concurrent training of strength and aerobic fitness for rowing and canoeing. Sports Med..

[27] De Luca V., Minganti C., Borrione P., Grazioli E., Cerulli C., Guerra E., Bonifacino A., Parisi A. (2016). Effects of concurrent aerobic and strength training on breast cancer survivors: A pilot study. Public Health.

[28] Dieli-Conwright C.M., Courneya K.S., Demark-Wahnefried W., Sami N., Lee K., Sweeney F.C., Stewart C., Buchanan T.A., Spicer D., Tripathy D. (2018). Aerobic and resistance exercise improves physical fitness, bone health, and quality of life in overweight and obese breast cancer survivors: A randomized controlled trial. Breast Cancer Res. BCR.

[29] Pagola I., Morales J.S., Alejo L.B., Barcelo O., Montil M., Oliván J., Álvarez-Bustos A., Cantos B., Maximiano C., Hidalgo F. (2020). Concurrent Exercise Interventions in Breast Cancer Survivors with Cancer-related fatigue. Int. J. Sports Med..

[30] Madeira R., Neiva H.P., Maia A., Esteves D. (2023). The Effect of Concurrent Training on Cancer Survivors: An Update of the Scientific Evidence. Physiother. Sports Inj..

[31] Cumpston M.S., McKenzie J.E., Welch V.A., Brennan S.E. (2022). Strengthening systematic reviews in public health: Guidance in the Cochrane Handbook for Systematic Reviews of Interventions. J. Public Health.

[32] Hunter J.E., Schmidt F.L., Le H. (2006). Implications of direct and indirect range restriction for meta-analysis methods and findings. J. Appl. Psychol..

[33] Garritty C., Hamel C., Trivella M., Gartlehner G., Nussbaumer-Streit B., Devane D., Kamel C., Griebler U., King V.J., Cochrane Rapid Reviews Methods Group (2024). Updated recommendations for the Cochrane rapid review methods guidance for rapid reviews of effectiveness. BMJ.

[34] Cohen J. (2013). Statistical Power Analysis for the Behavioral Sciences.

[35] Marques D.L., Neiva H.P., Marinho D.A., Marques M.C. (2023). Manipulating the Resistance Training Volume in Middle-Aged and Older Adults: A Systematic Review with Meta-Analysis of the Effects on Muscle Strength and Size, Muscle Quality, and Functional Capacity. Sports Med..

[36] Courneya K.S., McKenzie D.C., Mackey J.R., Gelmon K., Friedenreich C.M., Yasui Y., Reid R.D., Cook D., Jespersen D., Proulx C. (2013). Effects of exercise dose and type during breast cancer chemotherapy: Multicenter randomized trial. J. Natl. Cancer Inst..

[37] de Paulo T.R.S., Winters-Stone K.M., Viezel J., Rossi F.E., Aro B.L., Trindade A.C.A.C., Codogno J.S., Freitas Junior I.F. (2019). Comparing exercise responses to aerobic plus resistance training between postmenopausal breast cancer survivors undergoing aromatase inhibitor therapy and healthy women. Disabil. Rehabil..

[38] Dieli-Conwright C.M., Courneya K.S., Demark-Wahnefried W., Sami N., Norris M.K., Fox F.S., Buchanan T.A., Spicer D., Bernstein L., Tripathy D. (2021). Aerobic and resistance exercise improve patient-reported sleep quality and is associated with cardiometabolic biomarkers in Hispanic and non-Hispanic breast cancer survivors who are overweight or obese: Results from a secondary analysis. Sleep.

[39] Dieli-Conwright C.M., Courneya K.S., Demark-Wajmefried W., Sami N., Lee K., Buchanan T.A., Spicer D., Bernstein L., Mortimer J.E. (2018). Effects of Aerobic and Resistance Exercise on Metabolic Syndrome, Sarcopenia Obesity, and Circulating Biomarkers in Overweight 4or obese survivors of breast cancer: A randomized controlled trial. J. Clin. Oncol..

[40] Herrero F., San Juan A.F., Fleck S.J., Foster C., Lucia A. (2007). Effects of detraining on the functional capacity of previously trained breast cancer survivors. Int. J. Sports Med..

[41] Jones L.M., Stoner L., Baldi J.C., McLaren B. (2020). Circuit resistance training and cardiovascular health in breast cancer survivors. Eur. J. Cancer Care.

[42] An K.Y., Morielli A.R., Kang D.W., Friedenreich C.M., McKenzie D.C., Gelmon K., Mackey J.R., Reid R.D., Courneya K.S. (2020). Effects of exercise dose and type during breast cancer chemotherapy on longer-term patient-reported outcomes and health-related fitness: A randomized controlled trial. Int. J. Cancer.

[43] Mijwel S., Jervaeus A., Bolam K.A., Norrbom J., Bergh J., Rundqvist H., Wengström Y. (2019). High-intensity exercise during chemotherapy induces beneficial effects 12 months into breast cancer survivorship. J. Cancer Surviv. Res. Pract..

[44] Mostarda C., Castro-Filha J., Reis A.D., Sevílio M., Dias C.J., Silva-Filho A.C., Garcia J.B.S., do Desterro Nascimento M., Coelho-Junior H.J., Rodrigues B. (2017). Short-term combined exercise training improves cardiorespiratory fitness and autonomic modulation in cancer patients receiving adjuvant therapy. J. Exerc. Rehabil..

[45] Okumatsu K., Tsujimoto T., Wakaba K., Seki A., Kotake R., Yamauchi T., Hirayama S., Kobayashi H., Yamauchi H., Tanaka K. (2019). Effects of a combined exercise plus diet program on cardiorespiratory fitness of breast cancer patients. Breast Cancer.

[46] Reis A.D., Pereira P.T.V.T., Filha J.G.L.C., Rodrigues E.F., Laranjeira I.P., Ramallo B.T., Castro M.R., Rossi F.E., Júnior I.F.F., Garcia J.B.S. (2023). Effect of Combined Training on Body Image, Body Composition and Functional Capacity in Patients with Breast Cancer: Controlled Clinical Trial. Efeito do treinamento combinado na imagem corporal, composição corporal e capacidade funcional em pacientes com câncer de mama: Ensaio clínico controlado. Rev. Bras. De Ginecol. E Obstet. Rev. Da Fed. Bras. Das Soc. De Ginecol. E Obstet..

[47] Reis A.D., Pereira P.T.V.T., Diniz R.R., de Castro Filha J.G.L., Dos Santos A.M., Ramallo B.T., Filho F.A.A., Navarro F., Garcia J.B.S. (2018). Effect of exercise on pain and functional capacity in breast cancer patients. Health Qual. Life Outcomes.

[48] Thivat E., Thérondel S., Lapirot O., Abial C., Gimbergues P., Gadéa E., Planchat E., Kwiatkowski F., Mouret-Reunier M.A., Chollet P. (2010). Weight change during chemotherapy changes the prognosis in non metastatic breast cancer for the worse. BMC Cancer.

[49] Kroenke C.H., Chen W.Y., Rosner B., Holmes M.D. (2005). Weight, weight gain, and survival after breast cancer diagnosis. BMC Cancer.

[50] Schoenfeld B.J., Contreras B., Krieger J., Grgic J., Delcastillo K., Belliard R., Alto A. (2019). Resistance Training Volume Enhances Muscle Hypertrophy but Not Strength in Trained Men. Med. Sci. Sports Exerc..

[51] Hansen D., Abreu A., Ambrosetti M., Cornelissen V., Gevaert A., Kemps H., Piepoli M. (2021). Exercise intensity assessment and prescription in cardiovascular rehabilitation and beyond: Why and how: A position statement from the Secondary Prevention and Rehabilitation Section of the European Association of Preventive Cardiology. Eur. J. Prev. Cardiol..

[52] Loose B.D., Christiansen A.M., Smolczyk J.E., Roberts K.L., Budziszewska A., Hollatz C.G., Norman J.F. (2012). Consistency of the Counting Talk Test for Exercise Prescription. J. Strength Cond. Res..

[53] Ramos-Campo D.J., Andreu C.L., Martínez-Rodríguez A., Rubio-Arias J.Á. (2021). Effects of Resistance Circuit-Based Training on Body Composition, Strength and Cardiorespiratory Fitness: A Systematic Review and Meta-Analysis. Biology.

[54] Klassen O., Schmidt M.E., Ulrich C.M., Schneeweiss A., Potthoff K., Steindorf K., Wiskemann J. (2017). Muscle strength in breast cancer patients receiving different treatment regimes. J. Cachexia Sarcopenia Muscle.

[55] Schneider J., Schlüter K., Sprave T., Wiskemann J., Rosenberger F. (2020). Exercise intensity prescription in cancer survivors: Ventilatory and lactate thresholds are useful submaximal alternatives to VO2peak. Support Care Cancer.

[56] Meyer D., Pastor-Villaescusa B., Michel S., Hauner H., Hauner D. (2022). Associations between circulating obesity-related biomarkers and prognosis in female breast cancer survivors: A systematic review of observational data in women enrolled in lifestyle intervention trials. BMC Cancer.

[57] Kong L., Gao R. (2022). Aerobic exercise combined with resistance exercise training improves cardiopulmonary function and blood lipid of patients with breast cancer: A systematic review and meta-analysis. Medicine.

[58] Maginador G., Lixandrão M.E., Bortolozo H.I., Vechin F.C., Sarian L.O., Derchain S., Telles G.D., Zopf E., Ugrinowitsch C., Conceição M.S. (2020). Aerobic Exercise-Induced Changes in Cardiorespiratory Fitness in Breast Cancer Patients Receiving Chemotherapy: A Systematic Review and Meta-Analysis. Cancers.

[59] Groen W.G., Naaktgeboren W.R., van Harten W.H., van Vulpen J.K., Kool N., Sonke G.S., van der Wall E., Velthuis M.J., Aaronson N.K., May A.M. (2022). Physical Fitness and Chemotherapy Tolerance in Patients with Early-Stage Breast Cancer. Med. Sci. Sports Exerc..

[60] Bekhet A.H., Abdallah A.R., Ismail H.M., Genena D.M., Osman N.A., El Khatib A., Abbas R.L. (2019). Benefits of Aerobic Exercise for Breast Cancer Survivors: A Systematic Review of Randomized Controlled Trials. Asian Pac. J. Cancer Prev. APJCP.

[61] Fukushima T., Nakano J., Hashizume K., Ueno K., Matsuura E., Ikio Y., Ishii S., Morishita S., Tanaka K., Kusuba Y. (2021). Effects of aerobic, resistance, and mixed exercises on quality of life in patients with cancer: A systematic review and meta-analysis. Complement. Ther. Clin. Pract..

[62] Bower J.E., Ganz P.A., Desmond K.A., Bernaards C., Rowland J.H., Meyerowitz B.E., Belin T.R. (2006). Fatigue in long-term breast carcinoma survivors: A longitudinal investigation. Cancer.

[63] Rogers L.Q., Fogleman A., Trammell R., Hopkins-Price P., Spenner A., Vicari S., Rao K., Courneya K.S., Hoelzer K., Robbs R. (2015). Inflammation and psychosocial factors mediate exercise effects on sleep quality in breast cancer survivors: Pilot randomized controlled trial. Psycho-Oncol..

[64] de Roon M., May A.M., McTiernan A., Scholten R.J.P.M., Peeters P.H.M., Friedenreich C.M., Monninkhof E.M. (2018). Effect of exercise and/or reduced calorie dietary interventions on breast cancer-related endogenous sex hormones in healthy postmenopausal women. Breast Cancer Res. BCR.

[65] Kang D.W., Lee J., Suh S.H., Ligibel J., Courneya K.S., Jeon J.Y. (2017). Effects of Exercise on Insulin, IGF Axis, Adipocytokines, and Inflammatory Markers in Breast Cancer Survivors: A Systematic Review and Meta-analysis. Cancer Epidemiol. Biomark. Prev. A Publ. Am. Assoc. Cancer Res. Cosponsored By Am. Soc. Prev. Oncol..

[66] Meneses-Echávez J.F., Correa-Bautista J.E., González-Jiménez E., Schmidt Río-Valle J., Elkins M.R., Lobelo F., Ramírez-Vélez R. (2016). The Effect of Exercise Training on Mediators of Inflammation in Breast Cancer Survivors: A Systematic Review with Meta-analysis. Cancer Epidemiol. Biomark. Prev. A Publ. Am. Assoc. Cancer Res. Cosponsored By Am. Soc. Prev. Oncol..

[67] Ashton R.E., Tew G.A., Aning J.J., Gilbert S.E., Lewis L. (2020). Effects of short-term, medium-term and long-term resistance exercise training on cardiometabolic health outcomes in adults: Systematic review with meta-analysis. Br. J. Sports Med..

[68] Saco-Ledo G., Valenzuela P.L., Ruilope L.M., Lucia A. (2022). Physical exercise in resistant hypertension: A systematic review and meta-analysis of randomized controlled trials. Front. Cardiovasc. Med..

