# Supplementary material for: Efficacy of Concurrent Training in Breast Cancer Survivors: A Systematic Review and Meta-Analysis of Physical, Psychological, and Biomarker Variables
Source: Healthcare (Basel). 2024 Dec 27;13(1):33. doi: 10.3390/healthcare13010033 (PMC11719466; doi:10.3390/healthcare13010033)
Supplement: Supplementary file 1 [file healthcare-13-00033-s001.zip › Supplementary Figure S1 - Risk of Bias.pdf]

	Random sequence generation (selection bias)	Allocation concealment (selection bias)	Blinding of participants and personnel (performance bias): All outcomes	Blinding of outcome assessment (detection bias): All outcomes	Incomplete outcome data (attrition bias): All outcomes	Selective reporting (reporting bias)	Other bias
An 2020	+	+	+	+	+	+	+
Courneya 2013	+	+	−	?	+	+	+
de Paulo 2019	+	+	−	−	+	+	+
Dieli-Conwright 2018	+	+	+	+	+	+	+
Dieli-Conwright 2021	+	+	+	+	+	+	+
Jones 2020	?	+	+	+	+	+	+
Mijwel 2019	+	+	?	+	+	+	+
Mostarda 2017	−	?	?	+	+	+	+
Okumatsu 2019	−	−	?	?	+	+	+
Pagola 2020	+	+	+	+	+	+	+
Reis 2018	−	+	+	+	+	+	+
Reis 2023	+	+	+	+	+	+	+

Figure S1. Judgments about each risk-of-bias item for each included study: + indicates low risk, ? indicates unclear risk, − indicates high risk.
