# Supplementary material for: Efficacy of Concurrent Training in Breast Cancer Survivors: A Systematic Review and Meta-Analysis of Physical, Psychological, and Biomarker Variables
Source: Healthcare (Basel). 2024 Dec 27;13(1):33. doi: 10.3390/healthcare13010033 (PMC11719466; doi:10.3390/healthcare13010033)
Supplement: Supplementary file 1 [file healthcare-13-00033-s001.zip › Supplementary Table S1.pdf]

Table S1. Characterization of the population included in the systematic reviews included

Authors	Years	Sample size, N	Participant characteristics	Intervention time	Location
[27]	2016	20<br>IG=10 (50.2 ± 9.7)<br>CG=10 (46.0 ± 2.8)	Breast Cancer (mastectomy; no engagement in any formal exercise programs for at least 6 months; medical clearance to physical activity; the absence of musculoskeletal disturbances that could limit participation in the exercise training program)	Conclusion of all cancer related treatments at least 6 months previously	Italy
[29]	2020	23<br>IC=13 (51 ± 6)<br>CG=10 (47 ± 7)	Breast Cancer (18-65 years old; no evidence of tumor recurrence or metastasis; ≤5 years after last anti-cancer treatment; score <45 in the PERFORM questionnaire of fatigue; signing the written informed consent)	≤5 years after last anti-cancer treatment	Spain
[36]	2013	301<br>STAN-aerobic-96 (49.2 ± 8.4)<br>HIGH-aerobic 120min-101 (50.1±8.8)<br>COMB- A+R- 104 (50.5 ± 9.4)	Breast Cancer (18 years or older; stage I-III; non pregnant; speaking English or French)	Initiating adjuvant chemotherapy	Canada
[38]	2021	97<br>IG= 49 (53.3 ± 10.4)<br>CG= 48 (52 ± 10.4)	Breast Cancer (stage I-III; nonsmokers; physically inactive; BMI ≥25.0 kg/m^2 or body fat >30%; waist circumference >88cm)	<6 months posttreatment for chemo- or radiation-therapy	USA
[39]	2018	97<br>IG= 49 (52.8 ± 10.6)<br>CG= 48 (53.6 ± 10.1)	Breast Cancer (stage I-III; nonsmokers; sedentary; BMI ≥25.0 kg/m^2 or body fat >30%; waist circumference >88cm)	<6 months posttreatment	USA

[37]	2018	36<br>IG=18 (63.6±7.2)<br>CG=18 (NS)	Breast Cancer (stage I-III; women who were 50 years and over; no participation in supervised exercise in the previous 6 months; no musculoskeletal injuries; clearance from physician to participate in physical training)	Postmenopausal undergoing aromatase inhibitor therapy	Brazil
[40]	2007	11<br>IC=11 (47 ± 7)	Breast Cancer (stage I-II; postmenopausal women; 40-60 years; physical activity level: walking ≤ than a total oof 30-60min two days week and performing no strenuous exercise such as running, cycling, swimming or resistance training; previous anticancer treatment consisting of surgery with axillary lymphadenectomy and both post-surgery radiotherapy and chemotherapy)	2-5 years post-treatment	Spain
[41]	2020	51<br>IG=26 (55.8 ± 7.2)<br>CG=25 (55.9 ± 7.1)	Breast Cancer (40-70 years old; previously diagnosed and treated)	Completed adjuvant treatment at least 8 weeks	New Zeeland
[42]	2020	301<br>STAN=96 (NS)<br>HIGH=101 (NS)<br>COMB=104 (NS)	Breast Cancer (Non-pregnant women ≥18 years old; Stage I-III)	Initiating chemotherapy	Canada
[43]	2019	240<br>RT-HIIT= 74 (52.7 ± 10.3)<br>AT-HIIT= 72 (54-4 ± 10.3)	Breast Cancer (stage I-III; aged 18-70 years)	Planned to received adjuvant chemotherapy	Sweden

		CG=60 (52.6 ± 10.3)			
[44]	2017	18<br>IG=9 (NS)<br>CG=9 (NS)	Breast Cancer (stage I-III; 30-59 years; have undergone treatment within the first 6 months after surgery; physically inactive; normal left ventricle ejection fraction)	Receiving adjuvant radiotherapy, chemotherapy, or hormone therapy	Brazil
[45]	2019	32<br>IC=21 (51 ± 6)<br>CG=11 (53 ± 5)	Breast Cancer (20-75 years old; receiving an aromatase inhibitor or tamoxifen at least 1 year; no medical condition that prohibit participation in exercise plus diet program; experiencing various side effects after taking an endocrine therapy)	Survivor at least 1 year after surgery	Japan
[46]	2023	26<br>IG =13 (46.9 ± 7.4)<br>CG=13 (51.8 ± 12.5)	Breast Cancer (30-59 years old; not having performed physical training for the least 6 months; no diagnosis of mental or psychological disorders; able to communicate verbally; no motor restrictions; no pregnant or lactating; having previous release)	Undergoing treatment	Brazil
[47]	2018	30<br>IG=15 (47.64 ± 7.60)<br>CG=15 (45.79 ± 8.14)	Breast Cancer (had not engaged in physical over the previous 6 months)	Undergoing treatment	Brazil

CG = Control Group; IG = Intervention Group; NS = Not specified; AT = aerobic training, RT= resistance training; HIIT= high-intensity interval training; STAN=Aerobic exercise- 25-30min; HIGH= Aerobic exercise-50-60min; COMB= Aerobic and resistance training.
