# Supplementary figures and images for: Efficacy of Concurrent Training in Breast Cancer Survivors: A Systematic Review and Meta-Analysis of Physical, Psychological, and Biomarker Variables
Source: Healthcare (Basel). 2024 Dec 27;13(1):33. doi: 10.3390/healthcare13010033 (PMC11719466; doi:10.3390/healthcare13010033)

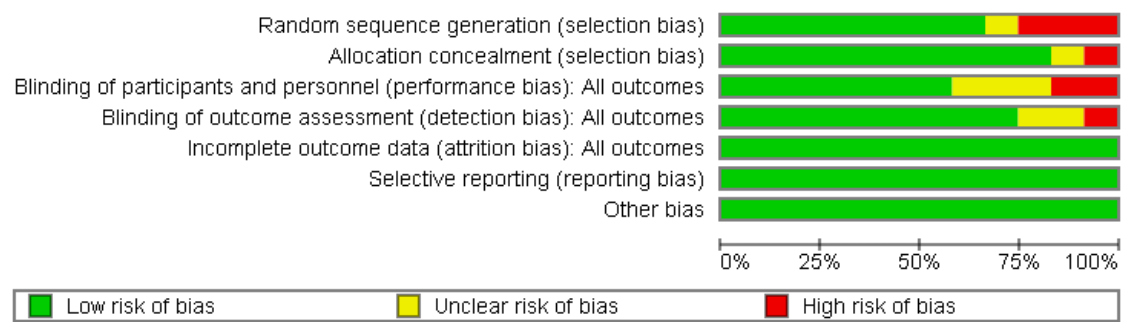

**Figure S2.** Risk-of-bias item presented as percentages across all included studies

Supplement: Supplementary file 1 [file healthcare-13-00033-s001.zip › Supplementary Figure S2 - Risk of Bias.pdf]
